# The quality of health information provided on web sites selling cannabis to consumers in Canada is poor

**DOI:** 10.1186/s12954-022-00691-9

**Published:** 2022-12-12

**Authors:** Jeremy Y. Ng, Umair Tahir, Nicholas Lum

**Affiliations:** Department of Health Research Methods, Evidence, and Impact, Faculty of Health Sciences, Michael G. DeGroote Centre for Learning and Discovery, McMaster University, Room 2112, 1280 Main Street West, Hamilton, ON L8S 4K1 Canada

**Keywords:** Cannabis (medical or recreational), Quality of information, Consumer health information, Information assessment, DISCERN

## Abstract

**Background:**

Cannabis is used by millions of people for both medical and recreational purposes, and this use is even greater in jurisdictions where it is legalized, such as Canada. Online cannabis vendors have gained popularity for purchasing cannabis due to easy access and convenience to consumers. The objective of this study was to evaluate the quality of health information provided by web sites of cannabis vendors selling products to Canadian consumers and to further identify trends in the information provided.

**Methods:**

Six different searches were conducted on Google.ca, and the first 40 webpages of each search were screened for eligibility. A total of 33 unique web sites of cannabis vendors selling products to Canadian consumers were identified and included. The DISCERN instrument, which consists of 16 questions divided into three sections, was used to evaluate the quality of cannabis-related health information on these web sites.

**Results:**

Across the 33 web sites, the average of the summed DISCERN scores was 36.83 (SD = 9.73) out of 75, and the mean score for the overall quality of the publication (DISCERN question 16) was 2.41 (SD = 0.71) out of 5. Many of these web sites failed to discuss uncertainties in research evidence on cannabis, the impact of cannabis use on quality of life, alternatives to cannabis use, risks associated with cannabis use, and lacked references to support claims on effects and benefits of use.

**Conclusion:**

Our findings indicate that the quality of cannabis-related health information provided by online vendors is poor. Healthcare providers should be aware that patients may use these web sites as primary sources of information and appropriately caution patients while directing them to high-quality sources. Future research should serve to replicate this study in other jurisdictions and assess the accuracy of information provided by online cannabis vendors, as this was outside the scope of the DISCERN instrument.

## Background

Cannabis was used for medical and recreational purposes by approximately 200.4 million people worldwide in 2019, making it the third most commonly used psychoactive substance after alcohol and tobacco [1]. Some of the proposed medical applications of cannabis include improvements in pain, dystonia, cachexia, multiple sclerosis spasticity, seizures resulting from Dravet syndrome and Lennox-Gastaut syndrome, glaucoma, and long-term use of opioids and opiate withdrawal [2]. Cannabis is also commonly used for cognitive issues such as anxiety/stress, post-traumatic stress disorder, depression, and insomnia [3]. Some risks associated with the use of cannabis include psychiatric manifestations such as psychosis, respiratory disease from long-term exposure, and low infant birth weight if cannabis is used during pregnancy [4, 5].

While recreational cannabis remains largely illegal on a global scale, medical cannabis has been legalized in countries such as Uruguay, the Netherlands, the United Kingdom, and over 30 states in the United States [6]. In Canada, cannabis was legalized for recreational use following the *Cannabis Act* in 2018, almost two decades after the legalization of medical cannabis in 2001 [7, 8]. Since then, the prevalence of cannabis use among Canadians has increased. In 2021, 25% of participants in the *Canadian Cannabis Survey* reported cannabis use compared to 22% in 2017, although this was a slight decrease from 2020 in which 27% reported cannabis use [9–11]. Due to this surge in interest, the number of online vendors selling cannabis products to consumers has also been rapidly growing. While each province and territory within Canada differ slightly with respect to their regulatory legal framework for cannabis sales, they all allow for the online purchase of cannabis provided that it is from either a government-operated or licensed private retailer [12]. This allows consumers the convenience of purchasing cannabis products online and having them delivered to them. Within their own jurisdictions, provinces and territories can determine how cannabis is sold, how stores are operated, and who can sell cannabis, while also having the ability to implement restrictions such as age and possession constraints [12]. For instance, the legal age is 18 in Alberta and 21 in Quebec, compared to 19 in all other provinces [12]. All cannabis products sold by retailers are mandated by the federal government to have health warning labels, but how the retailers operate, including the provision of health information, is determined by provincial or territorial jurisdiction [12]. All legal cannabis sold within Canada is required to be packaged with a health warning message and product-specific information such as the class of cannabis and cannabinoid content to provide consumers with the appropriate information to make informed decisions about usage [13]. Despite this legal framework and the licensing mandates regarding vendors, consumer interest in illegal and unlicensed online vendors has persisted. A qualitative study on the purchasing behavior of cannabis consumers noted that since the legalization of cannabis, the price of products sold by unlicensed vendors decreased and therefore such products have become more financially appealing due to affordability [14]. Unlicensed vendors were also noted as having better purchasing incentives such as sales and customer loyalty programs [14]. Therefore, it is not uncommon for consumers to purchase cannabis from illegal and unlicensed vendors rather than government-operated and licensed vendors.

With a greater number of cannabis consumers, it can be inferred that more individuals seek cannabis information online and may acquire their information from online vendors. A qualitative systematic review identified that patients using cannabis for pain reported conducting their own research online to find the best dispensaries and to understand which strains of cannabis and dosages would provide the best treatment for their specific condition [15]. Additionally, a survey about cannabis use for multiple sclerosis management found that patients most often relied on dispensaries for cannabis information [16]. In 2020, 69% of Canadians used the Internet to search for health information [17]. Online health information influences how consumers think, behave, and make decisions pertaining to their health, which is why the quality of such information is important [18, 19]. Youth, in particular, are frequently exposed to and influenced by information found online about cannabis [20].

To our knowledge, no studies have broadly examined the quality of the information provided by online cannabis vendors in the Canadian context specifically. Based on the existing literature, we hypothesized that the quality of health information provided by online cannabis vendors would be poor. Poor quality of information from such vendors can pose a risk to consumers, as online information may affect patient behavior and subsequent health-related decisions [21]. Given that an increasing number of Canadians are now seeking information surrounding cannabis and purchasing it via online vendors, the objective of the present study was twofold with respect to web sites of online cannabis vendors that ship to Canada: (1) to assess the quality of consumer health information and (2) to identify trends in information provision that contribute to the overall quality of these web sites for health information.

## Methods

### Search strategy

Consumer health information is sought primarily via Internet search engines, with Google holding the greatest market share and largely being the most popular among North American users [22]. We conducted preliminary searches to identify terms that consumers commonly use in place of cannabis (such as “marijuana” and “weed” [23]) and then devised a search strategy that would replicate common search queries of individuals seeking to buy cannabis online. This involved conducting six different searches on the first four pages of Google.ca (10 results per page, totaling 40 results per search term) based on the following search strategies: “buy marijuana online,” “buy cannabis online,” “buy weed online,” “purchase marijuana online,” “purchase cannabis online,” and “purchase weed online.” We based our search on the fact that most users search online using common words and short phrases, while staying away from any advanced search features; it is also known that most search engine users also do not browse past the first few result pages [24]. Searches were conducted on January 12, 2022. We used the incognito setting on the Google Chrome browser to ensure that previous browser search history would not influence the search results.

### Eligibility criteria and screening

After conducting the searches, we applied the following screening criteria, whereby web sites were only eligible if they were online cannabis vendors that shipped their cannabis product(s) to Canada. This includes both domestic and international vendors that ship their products to consumers in Canada. To mimic the searches that a typical consumer could conduct and the type of information that they would be exposed to online, vendors, regardless of their legal status, were included in our study. Exclusion criteria included: duplicate web sites, non-English-language web sites (or if bilingual, we only assessed the English component), web sites that were not online vendors of cannabis, web sites that were online vendors of cannabis but did not ship to at least one region in Canada, and web sites with inaccessible URLs. We also excluded vendor web sites if they declared that they would be ceasing their operations. Once all eligible web sites were identified, we assessed the quality of health information and evaluated trends in information provided.

### Data extraction and quality assessment of web site consumer health information

We pilot-tested data extraction, and the following information was collected: web site URL, vendor name, year vendor was established, type(s) of cannabis products sold (e.g., flower, buds, edibles, vapes, extracts), type(s) of cannabis accessories sold (e.g., rolling papers, bongs, vape batteries), and type(s) of non-cannabis products sold (e.g., mushrooms, LSD, clothing apparel). A quality assessment of the health information provided on each web site was also conducted using the DISCERN instrument. We met to compare our pilot data extractions, and discrepancies were resolved through discussion; the lead author (JYN) served as an arbitrator. Following this, we completed the data extraction and quality assessment for all remaining eligible web sites.

We chose to assess each web site using the DISCERN instrument (a standardized instrument developed by the British Library, National Health Service, and Oxford University), that has been validated and established as a reliable method to assess the quality of consumer health information present on a given treatment choice [25]. The DISCERN instrument consists of 15 distinct questions which examine a particular publication on the reliability and quality of the information provided. Examples of such information include descriptions of benefits and risks to treatment, references to information sources, and discussion of alternative treatment choices. Each web site was individually examined and rated for 15 questions where they were scored on a scale of 1 to 5 based on the detailed criteria provided by the DISCERN instrument [25]. These scoring criteria provided by DISCERN are explained in detail by the developers of the instrument [25]. Once a web site had been evaluated for the 15 preliminary questions, it was assessed for its overall quality, question 16, based on its performance on the previous questions. The summed DISCERN score was calculated for each web site by adding the scores from questions 1 to 15. Additionally, the mean and standard deviation were calculated for each of the 16 questions by aggregating the scores determined across the total number of included web sites.

Following the completion of data extraction and quality assessment, all authors met to compare and discuss scores. Discrepancies arising from misinterpretation of the data were resolved without unduly modifying legitimate discrepancies between assessors. In addition to presenting the general characteristics of eligible web sites, we present the means and standard deviations associated with each DISCERN question across web sites, as well as the total DISCERN score for each individual web site.

## Results

### Search results

**Fig. 1:**
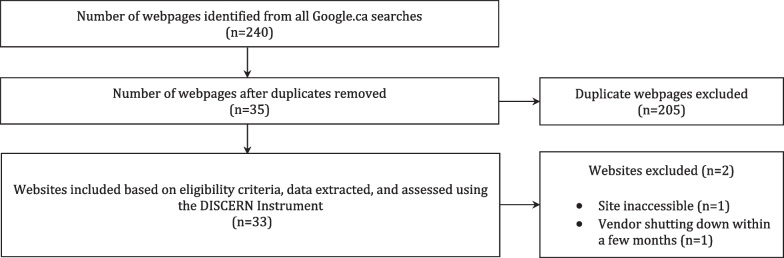
web information search strategy and assessment flowchart

Our search using Google.ca yielded a total of 240 webpages from the first 40 search results of each search strategy used, which was narrowed down to 35 unique web sites after removing duplicates. A further two web sites were excluded, as one had an inaccessible URL (*n* = 1) and another declared that their operations would cease in the coming months (*n* = 1). In total, 33 online vendor web sites were included based on the aforementioned eligibility criteria, underwent data extraction, and were assessed using the DISCERN instrument (Fig. 1).

### General characteristics of eligible web sites

Nine out of 33 (27%) web sites exclusively sold cannabis products, such as flowers, edibles, vapes, concentrates, and those topically applied. Twenty-four web sites (73%) sold cannabis accessories (e.g., rolling papers, bongs, pipes), and nine web sites also sold non-cannabis products (e.g., mushrooms, LSD, and clothing apparel). Eighteen web sites (55%) appeared in more than one search strategy. Information about the year in which the vendor was established was only available for nine web sites (27%), with the majority being established between 2017 and 2020 (*n* = 8), and the remainder being established before 2010 (*N* = 1). Common features among many web sites were that they contained “About Us” and blog pages. The “About Us” pages were commonly used to provide information including the aims and goals of the online vendor, while the blog pages often contained news and educational information about cannabis use, strains, effects, and safety. The general characteristics of all eligible web sites assessed using the DISCERN instrument, listed in alphabetic order by vendor name, are provided in Table 1.

**Table 1:** General characteristics of eligible web sites

web site name	URL	Year vendor established	Types of cannabis products sold by the vendor (i.e., edibles, vapes, teas, creams, etc.)	Types of cannabis accessories sold by the vendor (i.e., rolling papers, bongs, etc.)	Types of non-cannabis products sold by the vendor	Appeared in more than one search?
BC Cannabis Stores	https://www.bccannabisstores.com/	2018	Flower, edibles, vapes, extracts, topicals	Bongs, pipes, rolling papers, vape batteries	None	No
Budstars	https://budstars.com/	NR	Flowers, edibles, concentrates, oils, tinctures, topicals, capsules, and CBD	None	Mushrooms	Yes
Canna Cabana	https://cannacabana.com/	2009	Flower, vape cartridges, extracts, edibles, and drinks	Bongs, rigs, rolling papers, rolling trays	None	Yes
Canna Sweets	https://cannasweets.co/	NR	Flowers, concentrates, edibles, vape, and CBD	Rolling papers, vape batteries	LSD, Mushrooms	No
Cannabis Kings	https://cannabis-kings.net/	NR	Flower, THC and CBD concentrates, edibles, and vapes	None	None	No
Cannabis NB	https://www.cannabis-nb.com/	NR	Dried flower, pre-rolled, vapes, cartridges, edibles, beverages, oils, and capsules	Bongs, pipes, vape batteries, rolling papers	None	No
Cannabis NL	https://shopcannabisnl.com/	NR	Flower, concentrates, edibles, topicals	None	None	No
Cannabis Yukon	https://cannabisyukon.org/	2018	Flower, pre-rolled, edibles, gel capsules, oil, and seeds	None	None	No
Cannabismo	https://cannabismo.org/	NR	Flowers, concentrates, edibles, tinctures, and topicals	Grinders, rolling papers, pipes, vape batteries	None	Yes
Canvas Cannabis	https://www.canvascannabis.ca/	2019	Flower, pre-rolls, vaporizers, concentrates, edibles, tinctures, topicals, creams, gel, bath fizz, and lubricant	Pipes, bongs, lighters, vape batteries, rolling papers	Clothing Apparel	Yes
Cheap Weed	https://cheapweed.io/	NR	Flowers and concentrates	None	None	Yes
Dutch Love	https://dutch.love/	First established in 2019 as Hobo Cannabis, changed name in 2020 to Dutch Love	Flower, pre-rolls, vaporizers, concentrates, edibles, topicals, seeds	Rolling papers, vape batteries, lighters	None	No
Ganja Express	https://www.ganjaexpress.to/	NR	Flower, concentrates, edibles, CBD, and vapes	None	Pet Products (cannabis-based)	No
Get Kush	https://getkush.io/	NR	Cannabis flower, cannabis seeds, edibles, concentrates, vapes, CBD oils, CBD capsules, creams, lotions, and balms	Vape batteries, pipes	None	Yes
Green Society	https://greensociety.cc/	NR	Flowers, vapes, concentrates, edibles, and capsules	Vape batteries, grinders, rolling papers, lighters	Merchandise	Yes
Herb Approach	https://herbapproach.org/	NR	Flowers, edibles, vapes, and concentrates	Vape batteries, rolling papers, grinders	None	Yes
Hunny Pot Cannabis	https://thehunnypot.com/	2019	Flowers, pre-rolls, vaporizers, concentrates, edibles, tinctures, topicals, CBD, and seeds	Bongs, pipes, rolling papers, batteries	Clothing Apparel	Yes
Just Cannabis	https://justcannabis.cc/	NR	Flower, pre-rolled, concentrates, edibles, vapes, CBD	Vape batteries, grinders,	Mushrooms	No
King Canna	https://www.kingcannacanada.com/	NR	Flower, edibles, concentrates, CBD, and creams	Vape batteries	Mushrooms, Pet Products	Yes
Little Leaf Cannabis Co	https://www.littleleafcannabisco.com/	NR	Flower, pre-rolls, edibles, vapes, beverages, concentrates, oils, capsules, and topicals	Bongs, pipes, vape batteries, rolling papers	None	No
Ontario Cannabis Store	https://ocs.ca/	2017	Flower, vapes, extracts (concentrates), capsules, oral sprays, edibles, drinks, creams, and lotions	Vape batteries, bongs, pipes, rigs, rolling papers, grinders	None	Yes
PEI Cannabis	https://peicannabiscorp.com/	NR	Dried flower, pre-rolled, edibles, beverages, concentrates, and ingested extracts	Rolling papers, vape batteries, grinders	None	Yes
Seed and Stone	https://www.seedandstone.com/	NR	Flowers, pre-rolls, vapes, concentrates, and edibles	Pipes, rolling papers, bongs, pipes, vape batteries	None	Yes
Sessions Cannabis	https://www.sessions.ca/	NR	Flower, pre-rolls, vaporizers, concentrates, edibles, and topicals	Pipes, rolling papers, lighters, bongs, vape batteries	None	Yes
Shoppers Drug Mart	https://cannabis.shoppersdrugmart.ca/	2019	Dried cannabis, edibles, cannabis oil, softgels, concentrates, cartridges, topicals	None	None	Yes
SQDC	https://www.sqdc.ca/	2018	Dried cannabis, pre-rolled, beverages, and extracts	Rolling papers	None	Yes
Tale of Two Strains	https://www.taleoftwostrains.ca/	NR	Flowers, concentrates, capsules, edibles, topicals, and tinctures	Vape batteries, rolling papers	None	Yes
THC Canada	https://www.thccanada.ca/	NR	Flower, pre-rolls, vaporizers, concentrates, edibles, tinctures, topicals, CBD, and seeds	Vape batteries, rolling papers, grinders	None	No
The Pot Shack	https://www.thepotshack.ca/	NR	Flower, pre-rolled, edibles, beverages, vapes, extracts, oils, tinctures, CBD, topicals	Pipes, rolling paper	None	No
Toke	https://tokeonline.co/	NR	Flower, edibles, and extracts	None	None	No
Weed Smart	https://weedsmart.co/	NR	Flower, concentrates, edibles, CBD, vapes	Vape batteries, rolling papers	Mushrooms	No
Weedsy Canada	https://weedsy.ca/	NR	Flower, edibles, CBD, extracts, vapes, tinctures, and topicals	None	None	No
Xpress Grass	https://xpressgrass.com/	NR	Flowers, edibles, vapes, extracts, CBD, topicals	Vape batteries, rolling papers,	None	Yes

*NR* Not reported

### DISCERN instrument ratings

Across the 33 web sites, the average of the summed DISCERN scores was 36.83 (SD = 9.73) out of 75. The highest scoring web site was *SQDC* (summed DISCERN score of 59.5/75), while the lowest scoring web site was *Dutch Love* (summed DISCERN score of 20.5/75). The mean score for the overall quality of the web site (DISCERN question 16) was 2.41 (SD = 0.71) out of 5. Considering that both the mean of the summed DISCERN scores and the mean score for the overall quality of the publication were low, the quality of consumer health information provided by online cannabis vendors was found to be generally poor. The DISCERN question with the highest mean score (mean = 4.02; SD = 1.18) was question 10 (i.e., “Does it describe the benefits of each treatment?”). Conversely, the DISCERN question with the lowest mean score (mean = 1.08; SD = 0.18) was question 12 (i.e., “Does it describe what would happen if no treatment is used?”). Of the 15 questions assessed, only four questions (questions 1–3, and 10) had a mean score greater than 3.00. DISCERN scores for all questions assessed on each web site are presented in Table 2, listed in descending order of summed DISCERN score.

**Table 2:** DISCERN instrument ratings

Section		SECTION 1 Is the publication reliable?								SECTION 2 How good is the quality of information on treatment choices?							SECTION 3 Overall Rating of the Publication		
DISCERN Question		1. Are the aims clear?	2. Does it achieve its aims?	3. Is it relevant?	4. Is it clear what sources of information were used to compile the publication (other than the author or producer)?	5. Is it clear when the information used or reported in the publication was produced?	6. Is it balanced and unbiased?	7. Does it provide details of additional sources of support and information?	8. Does it refer to areas of uncertainty?	9. Does it describe how each treatment works?	10. Does it describe the benefits of each treatment?	11. Does it describe the risks of each treatment?	12. Does it describe what would happen if no treatment is used?	13. Does it describe how the treatment choices affect overall quality of life?	14. Is it clear that there may be more than one possible treatment choice?	15. Does it provide support for shared decision-making?	16. Based on the answers to all of the above questions, rate the overall quality of the publication as a source of information about treatment choices	Standard Deviation of Overall Score (Q16)	DISCERN Score (Sum of Q1-Q15)
SQDC	https://www.sqdc.ca/	5.00	5.00	4.50	1.50	1.00	4.50	5.00	4.50	4.00	5.00	5.00	1.50	4.50	3.50	5.00	4.00	0.00	59.50
Just Cannabis	https://justcannabis.cc/	5.00	4.50	4.50	4.50	4.00	3.50	4.50	4.00	5.00	5.00	4.00	1.00	2.50	2.50	2.00	3.50	0.00	56.50
Cannabis Kings	https://cannabis-kings.net/	3.50	3.50	3.50	5.00	5.00	3.50	5.00	5.00	5.00	5.00	5.00	1.00	1.50	1.50	1.00	4.00	0.00	54.00
Shoppers Drug Mart	https://cannabis.shoppersdrugmart.ca/	4.00	4.50	4.50	4.00	1.00	4.50	1.00	2.00	5.00	5.00	5.00	1.50	2.50	2.00	4.50	3.50	0.00	51.00
Ontario Cannabis Store	https://ocs.ca/	5.00	4.50	4.50	3.00	1.00	2.50	4.50	3.00	4.50	5.00	5.00	1.00	2.00	2.00	1.00	3.00	0.00	48.50
Seed and Stone	https://www.seedandstone.com/	4.50	5.00	3.50	4.00	5.00	3.00	3.00	1.00	4.00	4.50	2.50	1.00	2.00	2.50	1.00	3.00	0.00	46.50
Canvas Cannabis	https://www.canvascannabis.ca/	5.00	4.00	4.00	3.00	1.00	3.00	5.00	1.00	4.00	4.50	2.50	1.00	2.50	3.00	1.00	3.00	0.00	44.50
Get Kush	https://getkush.io/	3.00	4.00	3.50	3.00	4.00	3.00	1.50	3.00	3.00	4.50	3.50	1.50	2.50	2.50	1.50	3.00	0.00	44.00
BC Cannabis Stores	https://www.bccannabisstores.com/	5.00	4.50	4.50	3.00	1.00	4.00	1.50	1.00	4.00	3.00	5.00	1.00	1.00	2.00	1.00	3.00	0.00	41.50
Weed Smart	https://weedsmart.co/	3.00	3.00	4.00	3.00	1.00	4.00	1.00	1.50	4.00	5.00	5.00	1.00	1.00	2.00	3.00	3.00	0.00	41.50
Budstars	https://budstars.com/	3.00	3.50	3.50	3.00	2.00	2.50	2.00	3.00	4.00	5.00	1.50	1.00	2.00	2.00	1.00	2.50	0.00	39.00
Cannabis NB	https://www.cannabis-nb.com/	5.00	4.50	4.00	1.00	1.00	3.50	1.50	1.50	3.50	4.00	5.00	1.00	1.00	1.50	1.00	2.50	0.70	39.00
Cannabis Yukon	https://cannabisyukon.org/	3.00	3.00	4.50	2.50	1.00	3.00	3.00	3.00	3.00	3.00	5.00	1.00	1.00	2.00	1.00	2.25	0.35	39.00
Xpress Grass	https://xpressgrass.com/	2.50	2.50	3.50	4.00	4.00	2.50	3.00	3.00	3.00	4.50	1.00	1.00	1.00	1.00	1.00	2.00	0.00	37.50
Hunny Pot Cannabis	https://thehunnypot.com/	5.00	4.50	3.00	1.00	4.00	2.50	1.00	1.00	3.00	4.00	1.50	1.50	2.00	1.50	1.00	2.50	0.00	36.50
Green Society	https://greensociety.cc/	4.00	4.50	3.00	1.50	3.00	1.50	3.00	1.00	2.00	5.00	1.00	1.00	2.00	2.00	1.00	2.00	0.00	35.50
Weedsy Canada	https://weedsy.ca/	3.50	4.00	2.50	3.00	4.00	2.00	2.00	1.50	2.00	4.00	1.00	1.00	1.50	2.00	1.00	2.00	0.00	35.00
King Canna	https://www.kingcannacanada.com/	3.50	3.50	2.50	1.00	4.00	3.00	1.00	1.00	2.50	4.50	2.50	1.00	1.50	2.00	1.00	2.75	0.35	34.50
PEI Cannabis	https://peicannabiscorp.com/	5.00	4.50	3.50	1.00	1.00	3.00	1.00	1.50	2.00	3.00	2.00	1.00	1.00	1.50	3.00	2.25	0.35	34.00
Cannabis NL	https://shopcannabisnl.com/	3.50	4.00	3.00	1.50	1.00	2.50	4.00	1.00	2.50	3.00	1.50	1.00	1.50	2.50	1.50	2.25	0.35	34.00
Sessions Cannabis	https://www.sessions.ca/	2.00	2.50	3.00	2.00	4.00	2.00	2.00	1.00	3.00	4.50	1.50	1.00	2.00	1.50	1.00	2.00	0.00	33.00
Toke	https://tokeonline.co/	4.50	4.00	3.00	1.00	1.00	2.00	1.00	1.00	4.00	4.00	1.50	1.00	1.00	2.00	1.50	2.00	0.00	32.50
Herb Approach	https://herbapproach.org/	4.50	4.50	2.50	1.00	1.00	2.00	1.00	1.00	3.00	5.00	1.00	1.00	1.50	2.00	1.00	2.00	0.00	32.00
Canna Cabana	https://cannacabana.com/	2.00	2.00	3.00	1.00	3.00	1.50	1.00	3.00	3.00	3.00	2.50	1.00	2.00	2.50	1.00	2.00	0.00	31.50
Tale of Two Strains	https://www.taleoftwostrains.ca/	4.50	4.50	1.50	1.00	1.00	1.50	1.00	1.00	3.50	5.00	1.00	1.00	1.00	2.00	1.00	2.00	0.00	30.50
Cannabismo	https://cannabismo.org/	3.50	3.50	1.50	1.00	1.00	2.00	1.00	1.50	2.00	5.00	1.50	1.50	1.50	2.00	1.00	2.00	0.00	29.50
Cheap Weed	https://cheapweed.io/	3.00	3.50	2.50	1.00	3.00	1.50	1.00	1.00	1.50	4.50	1.00	1.00	1.50	2.00	1.00	2.00	0.00	29.00
Ganja Express	https://www.ganjaexpress.to/	4.00	4.50	2.00	1.00	1.00	2.00	1.00	1.00	1.50	4.00	1.00	1.00	1.50	1.50	1.00	2.00	0.00	28.00
Canna Sweets	https://cannasweets.co/	3.50	3.50	1.50	1.00	1.00	1.50	1.00	1.00	1.50	5.00	1.00	1.00	1.00	1.50	1.00	1.75	0.35	26.00
THC Canada	https://www.thccanada.ca/	2.50	2.50	2.00	2.00	3.00	1.50	1.00	1.00	1.50	2.00	1.00	1.00	1.00	1.50	1.50	2.00	0.00	25.00
Little Leaf Cannabis Co	https://www.littleleafcannabisco.com/	4.50	4.00	1.50	1.00	1.00	2.00	1.00	1.00	1.00	1.00	1.00	1.00	1.00	1.50	1.00	1.00	0.00	23.50
The Pot Shack	https://www.thepotshack.ca/	3.00	3.00	1.50	1.00	3.00	1.50	1.00	1.00	1.00	1.50	1.00	1.00	1.00	1.50	1.00	1.25	0.35	23.00
Dutch Love	https://dutch.love/	2.50	3.00	1.50	1.00	1.00	2.00	1.00	1.00	1.00	1.50	1.00	1.00	1.00	1.00	1.00	1.50	0.00	20.50
TOTAL means		3.79	3.82	3.06	2.08	2.21	2.56	2.05	1.79	2.95	4.02	2.44	1.08	1.62	1.94	1.44	2.41	0.09	36.83
TOTAL standard deviations		0.97	0.79	1.04	1.25	1.45	0.90	1.43	1.16	1.21	1.18	1.64	0.18	0.74	0.53	1.00	0.71	0.18	9.73

### Trends identified across resources assessed

#### Section 1: Is the publication reliable? (Questions 1–8)

Questions 1 and 2 assessed the aims of the web site, specifically inquiring whether the aims were stated clearly and whether they were achieved, respectively. The mean score for question 1 was 3.79 (SD = 0.97) and the mean score question 2 was 3.82 (SD = 0.79). web sites scoring higher in these questions usually had an “About Us” section, which stated clear and specific aims and values of the vendor, in addition to other general information such as the location of the distributor and target populations.

Question 3 assessed whether the information provided on the web site was relevant to cannabis consumers. Twenty-one out of 33 web sites (64%) received a score of 3 or greater on this domain, and the question received a mean score of 3.06 (SD = 1.04). web sites receiving a higher score provided information that was relevant to the needs of cannabis consumers, such as information about strains, dosing, methods of use, effects, as well as information on which patients with preexisting conditions may benefit from cannabis use.

Question 4 assessed whether the information provided on the web site was supported by external sources. Generally, most web sites rated poorly, with a mean score of 2.08 (SD = 1.25). web sites scoring in the 3 to 5 range provided citations embedded in the text and/or a complete reference list at the end of the information sections. Question 5 assessed if it was clear when the information used or reported on the web site was created. Fourteen out of 33 web sites (42%) scored 3 or greater, and the mean score was 2.21 (SD = 1.45). Generally, most web sites with blog posts containing information on cannabis provided dates of publication. Question 6 assessed whether the information provided on the web site was balanced and unbiased. The mean score for this question was 2.56 (SD = 0.90), and web sites scoring 3 or higher (39%) presented information in an objective manner, referenced multiple sources of information (peer-reviewed, governmental, or health information sites), and provided a balanced discussion of both the benefits and harms of cannabis use. Poorly scoring web sites tended to provide information in a sensational or promotional manner, or lacked sufficient information on cannabis products in general. Question 7 assessed whether the web site provided details of additional sources of support and information. Although the mean score for this domain was low (mean = 2.05 SD = 1.43), web sites scored highly if they suggested further readings and sources where readers could learn more in-depth about the cannabis topics presented on the web site.

Lastly, question 8 evaluated whether the web site referred to areas of uncertainty with respect to cannabis products. Only nine web sites (27%) scored 3 or higher on this question (mean = 1.79; SD = 1.16), as they referred to specific areas where further cannabis research is required, explicitly identified which claims are supported through limited evidence, and/or mentioned the variable effects of cannabis use.

#### Section 2: How good is the quality of information on treatment choices? (Questions 9–15)

Question 9 assessed whether the web site described how each of the listed cannabis products works, and 19 out of 33 (58%) web sites received a score of 3 or greater. Most web sites scoring highly on this question had a “cannabis education,” “learn about cannabis,” or other blog pages which provided information on how cannabis works on the body to produce its effects. The endocannabinoid system and neurotransmitter action were frequently discussed across many web sites.

Questions 10 and 11 assessed whether the web site correctly identified the benefits and the risks associated with cannabis use, respectively. Twenty-nine web sites (88%) adequately (score of 3 or greater) described the benefits of cannabis use (mean = 4.02; SD = 1.18); in contrast, only 10 (30%) web sites adequately described risks (mean = 2.44; SD = 1.64). web sites scoring poorly on both these questions identified limited to no benefits or risks, provided vague and general disclaimers, or often lacked information on the effects of cannabis use in general.

The remaining four questions in this section (questions 12 to 15) each scored poorly, all receiving mean scores less than 2. These questions assessed, respectively, whether the web site (i) described what would happen if no cannabis was used (question 12; mean = 1.08; SD = 0.18); (ii) described how the treatment choices affect overall quality of life (question 13; mean = 1.62; SD = 0.74); (iii) made it clear that there may be more than one possible treatment choice (question 14; mean = 1.94; SD = 0.53); and (iv) provided support for shared decision-making (question 15; mean = 1.44; SD = 1.00). As evident by these mean scores, most web sites were lacking in these areas and received scores ranging between 1 and 2.

#### Section 3: Overall rating of the web sites (Question 16)

Based on questions 1 to 15, question 16 evaluated the overall quality of the web site as a source of information about cannabis. Most web sites were determined to be poor in overall quality, as 70% received an overall score lower than 3, and the mean overall score of all web sites was 2.41 (SD = 0.71). Among other factors assessed, these low scores can be attributed mainly to the lack of discussion on uncertainties in research evidence on cannabis, quality of life, alternatives to cannabis use, and shared decision-making, along with a lack of references to the literature and provision of additional sources of support.

## Discussion

The present study used the DISCERN instrument to assess the quality of consumer health information provided by 33 web sites of vendors selling cannabis to consumers in Canada. Although online vendors facilitate easier access and convenience to individuals wishing to purchase cannabis, the quality of health information on these web sites was generally found to be low. Many of these web sites failed to discuss uncertainties surrounding the research evidence about cannabis, the impact of cannabis use on quality of life, alternatives to cannabis use, risks associated with cannabis use, and lacked references to support claims on effects and benefits of use. These findings should warrant concern among HCPs and researchers, considering that patients may use these vendors' web sites as primary sources of information informing their decision to purchase/consume cannabis. Physicians and other healthcare providers (HCPs) should be aware of these findings and appropriately caution patients who express interest in cannabis use.

A number of reasons may explain why the quality of consumer health information found on most web sites we assessed was low. First and foremost, these web sites were commercial in nature; vendors are motivated to present information in a sensational rather than objective manner, aiming to persuade consumers to buy their products. Naturally, this results inthe provision of biased and unbalanced information, with a greater focus on the benefits of cannabis use and less focus on the risks, as evident by the present study's results, along with other published studies [26, 27]. Presenting information that could potentially detract consumers from buying products, such as the side effects of cannabis use, is not in the interest of many online vendors [28]. Additionally, as evident by the aims and goals provided by many of these web sites, they are primarily concerned with selling their products as opposed to educating consumers [29]. In our study particularly, web sites scored poorly across multiple questions as they lacked essential information on cannabis topics including risks of treatment, impact on patient quality of life, and uncertainties in research, among others. Moreover, the research of cannabis is arguably an emerging field that has been given considerably more attention over recent years; therefore, clear information on the risks and benefits of its use may not be easily found by many web site owners. Furthermore, web site owners may lack the expertise in interpreting and providing health-related information [30–32]. There is also a significant amount of misinformation present online about cannabis [33], and web site owners may also be influenced by this especially if they lack the necessary training to read and interpret the peer-reviewed literature or other sources providing reliable, but complex, information.

### Comparative literature

This is the first study to broadly evaluate the quality of health information provided by online cannabis vendors selling to Canadian consumers, with no restrictions on health conditions or purpose of use. Ng et al. recently used the DISCERN instrument to evaluate the quality of web-based consumer health information at the intersection of cannabis and pain [34]. Although the averaged DISCERN scores were found to be higher than those in our study, the quality of health information in this area was still concluded to be poor. Considering that cannabis is commonly used by consumers for a wide range of conditions other than pain, it is important to broadly evaluate the quality of consumer health information without excluding other diseases/conditions. Additionally, that study included health portal, professional, cannabis news, non-profit, and commercial web sites from the Netherlands, the United States, and Canada, whereas our focus was specifically on commercial web sites selling cannabis products to Canadian consumers.

Similar studies have been conducted in jurisdictions other than Canada, primarily in the United States. In 2014, Boatwright et al. evaluated the quality of medical marijuana claims on popular web sites determined by online marketing tools, in which they found that 76% of claims made by web sites were inaccurate and were based on low-quality evidence [35]. In comparison to the objectives of the present study, Boatwright et al., evaluated the accuracy and quality of only three medical cannabis claims on each web site, as opposed to assessing the entire web site. Three other studies from the United States (Luc et al., Cavazos-Rehg et al., and Kurger et al.) analyzed the content provided by online cannabis retailers, and concluded that many dispensaries made unsubstantiated claims about the benefits of cannabis for various conditions, such as nausea, depression, and anxiety [36–38]. Aligning with the findings from our study, Luc et al. and Kruger et al. also reported that there was limited mention of potential side effects or risks associated with cannabis use [36, 38]. Moreover, a study from the United Kingdom found that much of the online information about medical cannabis would raise unrealistic expectations of benefits and downplay potential side effects [26]. In agreement with this finding, one study noted that these effects were magnified by commercial web sites compared to other sources of online information (e.g., government, health portal, non-profit), indicating the biases associated with commercial interests [26]. Across other forms of media such as news outlets and online discussion forums, the quality and accuracy of cannabis-related health information, as well as reporting of risks, was also found to be poor [26, 39–41]. It is worth mentioning that these aforementioned studies did not use the DISCERN instrument, which in addition to quality, assesses the reliability of consumer health information, as opposed to accuracy.

### Implications and future directions

The low quality of health information provided by online cannabis vendors poses potential health risks to consumers. We found that crucial information such as warnings, adverse effects, and/or contraindications associated with the use of cannabis was lacking on many web sites. In addition to this, studies conducted in other jurisdictions suggest that many of the online health claims surrounding cannabis are either unsubstantiated, low in accuracy, and/or derived from a low level of evidence [35–40]. Together, these may lead to the misuse of cannabis and have major safety implications for consumers. Currently, promotions prohibitions exist under the *Cannabis Act and Cannabis Regulations* wherebystate that cannabis or cannabis products “cannot be promoted in a manner that is false, misleading or deceptive or that is likely to create an erroneous impression about its characteristics, value, quantity, composition, strength, concentration, potency, purity, quality, merit, safety, health effects or health risks” [42]. Despite this, many online vendorsappear to not abide by these standards.

Patients may bring up their interest in or their use of cannabis to HCPs, who should be aware of the low quality of cannabis-related health information provided by online vendors as highlighted by our findings. HCPs should appropriately caution patients about these findings and refer them to sources of high-quality information, such as the National Center for Complementary and Integrative Health [43]. This will ensure that patients are adequately informed prior to purchasing cannabis online and may ultimately guide purchasing and using behaviors. Unfortunately, another concern relates to HCPs’ training and education on the topic of cannabis, both in Canada and across other jurisdictions. As many HCPs lack the necessary knowledge to effectively counsel patients about the safe use of cannabis, they are often reluctant to discuss this as a therapeutic option with patients [44–46]. With the known and rapid increase in cannabis vendors and the low-quality information provided by them online, it is of urgent importance to adequately train HCPs and HCP students so that patients have a reliable provider of information to turn to for guidance. Public health agencies and those involved in cannabis-specific health policy may consider using the present study as a resource to inform HCPs and patients alike of the high likelihood of low-quality information being provided by online cannabis vendors.

Although our study evaluated the quality of cannabis-related health information provided by vendors that ship to Canada, and similar research has been conducted in the United States, it would be beneficial for further research to replicate this study in other jurisdictions where cannabis has been legalized in a similar manner, such as South Africa [47]. Moreover, one study identified that source credibility had no significant effect on consumers’ interpretation of the quality of online health information [48]. Future research should also evaluate the accuracy of information provided by online cannabis vendors, similar to the approach used by Boatwright et al. for medical cannabis, as this was not possible using the DISCERN instrument [35]. When doing this, a special focus should be placed on distinguishing between the type of sources used to support information (e.g., peer-reviewed literature versus blog posts written by non-experts), allowing for low-quality information and inaccuracies to be identified. Lastly, one study examined the implementation and effectiveness of online responsible vendor training for recreational marijuana in the United States, revealing that most employees were satisfied with the training and found it user-friendly [49]. Although this training was mainly focused on regulatory practices such as using the state’s inventory tracking system or checking for valid identifications, the authors suggest that cannabis-specific training on topics such as safety and dosing is a crucial future step. Such training may be beneficial for owners of online cannabis vendors in Canada as well as other jurisdictions, allowing them to incorporate important topics related to cannabis safety in online descriptions, thus research evaluating the effectiveness of such training would be of value. Lastly, the extent to which information provided online by vendors may influence consumers’ choice and usage behaviors of cannabis products is not well understood [36]. It is possible that misleading or inaccurate descriptions may cause consumers to misuse products in ways that may pose significant health risks. Therefore, future research should serve to study whether online cannabis information may have such effects on consumers.

### Strengths and limitations

One strength of the present study included the use of a validated and reliable instrument, DISCERN, to assess the quality of consumer health information about cannabis products. Another strength was the use of six different search terms on Google.ca, of which the first 40 search results were viewed for each (totaling 240 webpages), ensuring that the most frequently visited online cannabis vendors were captured and assessed. To our knowledge, this study is the first to assess the quality of health information provided by online cannabis vendors in Canada, with no restrictions to information on certain medical conditions.

One limitation to our methodology was that only web sites with English-language content were eligible, potentially excluding web sites in French (Canada’s second national language), among other languages. Additionally, considering that the Internet is constantly changing, we acknowledge that we only identified and assessed web sites at a certain snapshot of time. Therefore, if our study was replicated in the future, the content on many of these web sites may have changed, and different web sites (and the quality of information they provide) may appear in the search results. Further, an inherent limitation of the DISCERN tool is that while it can be used to assess whether references and additional sources of information are provided by web sites, it does not distinguish between the types of sources, such as peer-reviewed scientific literature versus blog posts, where the former would be deemed more credible in most cases and increase the quality of the information provided by the web site.

## Conclusion

Given a large number of cannabis users in Canada, purchasing cannabis from online vendors has gained increasing interest among consumers due to its easy accessibility and convenience. This study evaluates the quality of health information provided by web sites of cannabis vendors selling products to Canadian consumers. The DISCERN instrument was used to evaluate 33 web sites meeting the eligibility criteria. Our findings indicate that the quality of cannabis-related health information provided by online vendors is poor. Given that consumers may use these web sites as primary sources of information prior to purchasing cannabis, researchers and HCPs should be made aware of this; additionally, a need exists to create adequate training, so that HCPs can appropriately advise inquiring patients about the safety and efficacy of cannabis, and direct them to high-quality information resources. Future research should be directed at understanding the extent to which information provided by online vendors may influence consumer purchase and usage behaviors. It may also be of value to replicate this study across other jurisdictions and assess the accuracy of information provided by online cannabis vendors.

## Data Availability

All relevant data are included in this manuscript.

## Abbreviation

HCP: Healthcare provider
